# Stratified proportional win‐fractions regression analysis

**DOI:** 10.1002/sim.9570

**Published:** 2022-09-14

**Authors:** Tuo Wang, Lu Mao

**Affiliations:** ^1^ Department of Biostatistics and Medical Informatics, School of Medicine and Public Health University of Wisconsin‐Madison Madison Wisconsin

**Keywords:** composite endpoints, Lindeberg‐Feller condition, win ratio, proportionality assumption, U‐statistics

## Abstract

The recently proposed proportional win‐fractions (PW) model extends the two‐sample win ratio analysis of prioritized composite endpoints to regression. Its proportionality assumption ensures that the covariate‐specific win ratios are invariant to the follow‐up time. However, this assumption is strong and may not be satisfied by every covariate in the model. We develop a stratified PW model that adjusts for certain prognostic factors without setting them as covariates, thus bypassing the proportionality requirement. We formulate the stratified model based on pairwise comparisons within each stratum, with a common win ratio across strata modeled as a multiplicative function of the covariates. Correspondingly, we construct an estimating function for the regression coefficients in the form of an incomplete U‐statistic consisting of within‐stratum pairs. Two types of asymptotic variance estimators are developed depending on the number of strata relative to the sample size. This in particular allows valid inference even when the strata are extremely small, such as with matched pairs. Simulation studies in realistic settings show that the stratified model outperforms the unstratified version in robustness and efficiency. Finally, real data from a major cardiovascular trial are analyzed to illustrate the potential benefits of stratification. The proposed methods are implemented in the R package WR, publicly available on the Comprehensive R Archive Network (CRAN).

## 1 INTRODUCTION

Modern phase‐III clinical trials often combine death and certain nonfatal events as their primary efficacy endpoint.^1^, ^2^ The recent INfluenza Vaccine to Effectively Stop Cardio Thoracic Events and Decompensated Heart Failure (INVESTED; NCT02787044) trial, for example, compares the effects of two influenza vaccines in high‐risk cardiovascular (CV) patients on the composite of all‐cause death and cardiopulmonary hospitalization.^3^ To prioritize death over nonfatal events like hospitalization, Mao and Wang^4^ proposed a class of proportional win‐fractions (PW) regression models based on the pairwise‐comparison scheme of the two‐sample win ratio.^5^, ^6^, ^7^, ^8^, ^9^, ^10^, ^11^, ^12^, ^13^, ^14^, ^15^ This provides an alternative to the traditional Cox proportional hazards (PH) regression of time to the first event. Like the Cox model, the PW model also relies on a proportionality assumption, that is, that the covariate‐specific “win fractions” are proportional over time. This assumption is essential to the interpretation of the regression coefficients as the time‐invariant log‐win ratios.

Unlike the Cox model, however, the PW model lacks a stratification strategy.^16^ When patients tend to be more similar within groups defined by, for example, age, sex, and study center, adjusting for the groups will allow the treatment effect to be estimated more precisely. A direct way to do so is to include the group affiliations as covariates. This approach is non‐robust because it requires additional proportionality assumption on the group indicators. The PW model becomes invalid when any of its covariates fail to satisfy the assumption, in which case the regression coefficients become a censoring‐weighted mix of time‐dependent (log‐)win ratios (similarly to the Cox model under a faulty PH assumption^17^). Moreover, when the number of groups is large, setting them all as covariates can overburden and destabilize the model. Statistical considerations aside, clinicians may have substantive reasons to stratify patients as well. The aforementioned INVESTED trial, for example, recruited CV patients over multiple influenza seasons. Only those in the same season are clinically comparable as they are exposed to the same season‐specific viral strains.^3^ Similar comparability arguments justify stratified analysis by CV disease etiology, for example, ischemic versus non‐ischemic.^18^

In Cox‐type models, whether for a univariate event,^16^ recurrent events, or competing risks,^19^ stratification is typically done by modifying the “risk set” in a partial‐likelihood‐type estimating function so that only subjects in the same stratum are included. Despite the parallels, a stratified PW model does not follow immediately from either the stratified Cox model or unstratified PW model. First, the PW model does not contain a “baseline function” as Cox‐type models do that captures the between‐strata heterogeneity and that motivates the stratum‐specific risk set. Second, the estimating function for the PW model takes the unconventional form of a U‐statistic,^4^ which likely requires modification under stratification. Third, the inference in the unstratified model relied on large‐sample evaluation of U‐statistics.^4^, ^20^ The same strategy may become inadequate when the sample is divided into strata of much smaller sizes.

We develop a coherent framework to address these challenges in the specification, estimation, and inference of stratified PW models. In Section 2, we define the stratified model under a user‐specified rule of comparison, which permits prioritization of death over nonfatal events as does the standard PW model.^4^ Unlike the standard version, however, we consider only within‐stratum comparisons, similarly to the stratified two‐sample win ratio of Dong et al^11^ and stratified odds ratio of Mantel and Haenszel.^21^ The exclusion of between‐strata comparisons implicitly allows the “baseline” event rates to vary across strata. In keeping with the model, we construct an estimating function using a weighted sum of within‐stratum U‐statistics (a form of “incomplete U‐statistic”^22^). In Section 3, we develop two types of variance estimators depending on the number of strata relative to the sample size. When the number is small enough (eg, sex and race groups) to allow a fair‐sized cohort in each stratum, we derive the variances of the stratum‐specific U‐statistics by standard asymptotics. Then the variance of the estimating function follows as a weighted sum of the independent strata. In case of numerous strata with only sparse data within, we treat the stratum itself as the basic unit and apply the Lindeberg‐Feller central limit theorem to derive the variance of their weighted sum. This latter approach allows us to fit the PW model to matched pairs, which are essentially strata of two. In Section 4, we conduct simulation studies to assess the finite‐sample performance of the proposed methods in comparison with the unstratified PW model. As an illustration, a major cardiovascular trial is analyzed in Section 5 stratified by patient sex and age. Section 6 concludes the paper with some practical considerations and thoughts about future research.

## 2 MODEL AND ESTIMATION

### 2.1 Full data and model specification

Let D denote the survival time and $N_{D}(t)=I(D\leq t)$ the corresponding counting process, where I(·) is the indicator function. As in Mao and Wang,^4^ we assume that there are K distinct types of nonfatal events with counting processes denoted by $N_{1}(t),\ldots ,N_{K}(t)$, arranged in descending order of importance. In a standard application, K=1 so that $N_{1}(t)$ counts all relevant nonfatal events. Let $\bar{N}(t)=\{N(u):0\leq u\leq t\}$ denote the event history of a generic counting process N(·). Write $Y(t)=\left\{{\bar{N}}_{D}(t),{\bar{N}}_{1}(t),\ldots ,{\bar{N}}_{K}(t)\right\}$. Then Y(t) contains all outcome data collected up to time t. With τ denoting the maximum length of follow‐up, Y(τ) represents the “full” data of interest (without censoring). Let there be $L\in {\mathbb{Z}}^{+}$ strata. For all notation introduced above, we use subscripts li and lj to denote the corresponding values on the ith and jth subjects, respectively, in stratum l=1,…,L. Similarly to the standard PW model, we consider a general win indicator function, but only for within‐stratum comparisons^4^:

$$𝒲(Y_{li},Y_{lj})(t)=I(Y_{li}\;\text{wins against}\;Y_{lj}\;\text{up to time}\;t).$$

We require that 𝒲(·,·) satisfy the following three conditions for every t∈[0,τ]:

(A1) $𝒲(Y_{li},Y_{lj})(t)$ is a function of $Y_{li}(t)$ and $Y_{lj}(t)$ only;
(A2) $𝒲(Y_{li},Y_{lj})(t)+𝒲(Y_{lj},Y_{li})(t)=0\;\text{or}\;1;$
(A3) $𝒲(Y_{li},Y_{lj})(t)=𝒲(Y_{li},Y_{lj})(D_{li}\wedge D_{lj}\wedge t)$, where x∧y=min(x,y).

Remark 1 Condition (A1) ensures that a comparison made at t uses only information available at that time; (A2) means that the comparison is antisymmetric so that there is at most one winner; (A3) requires that the win‐loss status remain unchanged after either patient dies, avoiding complications caused by death as a competing risk.

A commonly used 𝒲 is the prioritized comparison proposed originally by Pocock et al^5^ for the two‐sample win ratio. Let T denote time to the first nonfatal event. Then, Pocock's win function can be written as

$${𝒲}_{P}(Y_{li},Y_{lj})(t)=I(D_{lj}<D_{li}\wedge t)+I(D_{lj}\wedge D_{li}>t,T_{lj}<T_{li}\wedge t).$$

It can be easily shown that ${𝒲}_{P}$ satisfies Conditions (A1)‐(A3) above. Other win functions that utilize recurrent events are studied by Mao et al.^23^ Instead of the first nonfatal event, for example, we can draw on the cumulative number of events experienced, with ties broken by time to the latest occurrence. The specific expression of this win function is given in the Supporting Information. Besides these prioritized comparisons, one can also focus on time to the first composite event, that is,

$${𝒲}_{TFE}(Y_{li},Y_{lj})(t)=I({\tilde{T}}_{lj}<{\tilde{T}}_{li}\wedge t),$$

where $\tilde{T}=D\wedge T$. This of course is less desirable as it uses fewer data and does not differentiate death from nonfatal events.

Let $Z={\left(Z_{1},\ldots ,Z_{p}\right)}^{T}$ denote a p‐dimensional vector of covariates, so $Z_{li}$ and $Z_{lj}$ are covariates for subjects li and lj, respectively. Following the standard PW model,^4^ we define the time‐dependent covariate‐specific win ratio in the lth stratum (l=1,…,L) by taking the quotient between the conditional win fractions, that is,

$${ℛ}_{l}\left(t|Z_{li},Z_{lj};𝒲\right)\equiv \frac{E\left\{𝒲(Y_{li},Y_{lj})(t)|Z_{li},Z_{lj}\right\}}{E\left\{𝒲(Y_{lj},Y_{li})(t)|Z_{li},Z_{lj}\right\}}.$$

Now, we model the stratum‐specific win ratio by

(1) $${ℛ}_{l}\left(t|Z_{li},Z_{lj};𝒲\right)=\exp\left\{\beta^{T}\left(Z_{li}-Z_{lj}\right)\right\}\;(l=1,\ldots ,L),$$

where $\beta ={\left(\beta_{1},\ldots ,\beta_{p}\right)}^{T}$ is a p‐dimensional regression parameter shared by all L groups. This means that, similarly to a stratified Cox model,^16^ the covariate effects are the same in each stratum (with possibly different baseline rates).

The main difference of (1) with the unstratified version^4^ is that only within‐stratum comparisons are modeled here. As a result, the coordinates of β are interpreted as the log‐win ratios associated with unit increases in the covariates *within each stratum*. For example, if the model is stratified by sex with a treatment indicator as the only covariate, then exp(β) is the common win ratio comparing the treatment to control within the male and female populations. While model (1) still requires that the within‐stratum win fractions be proportional over time (or equivalently, ${ℛ}_{l}\left(t|Z_{li},Z_{lj};𝒲\right)$ be constant with respect to t∈[0,τ]), the relationships of between‐strata win fractions are left unspecified. In other words, $E\left\{𝒲(Y_{li},Y_{l^{\prime }j})(t)|Z_{li},Z_{l^{\prime }j}\right\}/E\left\{𝒲(Y_{l^{\prime }j},Y_{li})(t)|Z_{li},Z_{l^{\prime }j}\right\}$ for $l\neq l^{\prime }$ can be any function of $Z_{li},Z_{l^{\prime }j}$, and t. Thanks to this feature, we can address the nonproportionality of a categorical predictor by using it as a stratifier.

Use SPW(𝒲) to denote the stratified PW model (1) under win function 𝒲. By derivation similar to that for the unstratified model,^4^ one can show that SPW$\left({𝒲}_{TFE}\right)$ is equivalent to the stratified Cox model on $\tilde{T}$ with log‐hazard ratios equal to the negative of the log‐win ratios, that is,

$$\lambda_{l}(t|Z_{li})=\exp(-\beta^{T}Z_{li})\lambda_{0l}(t),$$

where $\lambda_{l}(t|Z_{li})$ is the conditional hazard function of ${\tilde{T}}_{li}$ given $Z_{li}$ and $\lambda_{0l}(t)$ is an arbitrary (stratum‐specific) baseline hazard function. Likewise, we can show that SPW$\left({𝒲}_{P}\right)$ is implied by the “stratified” Lehmann model^4^

(2) $$P(D_{li}>s,T_{li}>t|Z_{li})=H_{l}{\left(s,t\right)}^{\exp(-\beta^{T}Z_{li})},$$

where $H_{l}(s,t)$ is a nonparametric (stratum‐specific) baseline joint survival function (with arbitrary between‐component correlation). For the recurrent‐event win function mentioned earlier, the SPW model holds under an extended Lehmann model (2) to all events involved. The details can be found in the Supporting Information. As in the unstratified case, joint models like (2) are sufficient but not necessary for the SPW in multivariate settings (see, eg, §S.1.2 of the online Supporting Information of Mao and Wang^4^).

### 2.2 Observed data and estimation

In practice, subjects are censored due to study termination or loss of follow‐up. As a result, the pairwise win/loss indicators may not be directly computable. Let C denote the censoring time and assume that C⊥Y|Z. This means that censoring is independent of all outcome events given the covariates. With X=D∧C, the observed data consist of

$$\{Y_{li}(X_{li}),X_{li},Z_{li}\},i=1,\ldots ,n_{l};l=1,\ldots ,L,$$

where $n_{l}$ is sample size of the lth stratum. Proceeding stratum‐wise along the lines of lemma 1 of Mao and Wang,^4^ we easily find that

(3) $$\frac{E\left\{𝒲(Y_{li},Y_{lj})(X_{li}\wedge X_{lj}\wedge t)|Z_{li},Z_{lj}\right\}}{E\left\{𝒲(Y_{lj},Y_{li})(X_{li}\wedge X_{lj}\wedge t)|Z_{li},Z_{lj}\right\}}=\exp\left\{\beta^{T}\left(Z_{li}-Z_{lj}\right)\right\}\;(l=1,\ldots ,L).$$

By (A1), the $\delta_{li,lj}(t)\equiv 𝒲(Y_{li},Y_{lj})(X_{li}\wedge X_{lj}\wedge t)$ are functions of the observed data. So we can use (3) to construct stratum‐specific pairwise residual processes

$$\begin{array}{lcr} M_{li,lj}(t|Z_{li},Z_{lj};\beta )=\delta_{li,lj}(t)-R_{li,lj}(t)\mu (Z_{li},Z_{lj};\beta ), & & \end{array}$$

where $R_{li,lj}(t)=\delta_{li,lj}(t)+\delta_{lj,li}(t)$ and

$$\mu \left(Z_{li},Z_{lj};\beta \right)=\frac{\exp\left\{\beta^{T}\left(Z_{li}-Z_{lj}\right)\right\}}{1+\exp\left\{\beta^{T}\left(Z_{li}-Z_{lj}\right)\right\}}.$$

Indeed, (3) can be shown to imply

(4) $$E\left\{M_{li,lj}(t|Z_{li},Z_{lj};\beta )|Z_{li},Z_{lj}\right\}=0\;(l=1,\ldots ,L).$$

Given (4), we can form an estimating function for β by integrating all pairwise residuals in each stratum with respect to some covariate weight function and then combining them together. Using $\overset{n_{l}}{\underset{i<j}{\sum }}\sum$ as a shorthand notation for $\overset{n_{l}-1}{\underset{i=1}{\sum }}\overset{n_{l}}{\underset{j=i+1}{\sum }}$, consider

(5) ∑l=1Lnl2−1∑i<jnl∑Zli−Zlj∫0τh^lt;Zli,Zlj;βMli,ljdt|Zli,Zlj;β=0,

where ${ĥ}_{l}(t;\cdot ,\cdot ;\beta )$ is some stratum‐specific real‐valued symmetric function converging uniformly in probability to some fixed function $h_{l}(t;\cdot ,\cdot ;\beta )$. The estimator $\hat{\beta }$ can be obtained by solving (5) using the Newton‐Raphson algorithm, which generally converges fast. Following conventions for the two‐sample win ratio^11^ as well as the Mantel‐Haenszel test, we recommend the simple weight ${ĥ}_{l}(t;\cdot ,\cdot ;\beta )\propto n_{l}/n$, where $n=\sum_{l=1}^{L}n_{l}$. Some heuristic justifications for this weight on efficiency grounds are provided in the Supporting Information.

### 2.3 Proportionality assumption assessment

Under the proportionality assumption, $M_{li,lj}(t|Z_{li},Z_{lj};\beta )$ has conditional mean zero for all t∈[0,τ] and l=1,…,L. This motivates us to use the score process

(6) U(t;β)=n−1∑l=1Lnl2−1nl∑i<jnl∑Zli−ZljMli,ljt|Zli,Zlj;β

to check the proportionality assumption. This is similar to the score process for the standard PW model^4^ except that it involves only within‐stratum scores (since model (1) requires only within‐stratum proportionality).

Specifically, we can check the proportionality of $Z_{j}$ by plotting ${\tilde{U}}_{j}(t;\hat{\beta })$ against t∈[0,τ], where ${\tilde{U}}_{j}(t;\hat{\beta })$ is the jth component of $U(t;\hat{\beta })$ standardized by the square root of the jth diagonal element of the variance estimator $\hat{\mathrm{Var}}\{U(\tau ;\hat{\beta })\}$ (obtained as a byproduct of the variance estimation in Section 3). When proportionality holds, ${\tilde{U}}_{j}(t;\hat{\beta })$ should behave like a (time‐rescaled) Brownian bridge with mean zero. Hence, any systematic trend might indicate violation of proportionality. As a rule of thumb, we accept proportionality if ${\tilde{U}}_{j}(t;\hat{\beta })$ is bounded between [−2,2].^4^

## 3 ASYMPTOTIC PROPERTIES AND VARIANCE ESTIMATION

We consider two set‐ups for variance estimation, one with a fixed L, the other with L→∞. The former is applicable when the number of strata is small relative to the sample size, such as in stratified analysis by sex. The latter is more appropriate when there are numerous small strata, such as in analysis of matched pairs.

### 3.1 Variance under finite strata (L<∞)

Given a sufficient number of patients in each stratum as n→∞, we can treat each stratum as an unstratified sample and apply the Hoeffding decomposition^20^ to linearize each of the stratum‐specific U‐statistics in (5). This gives rise to a linearized form of the whole estimating function. More formally, write $O_{li}=\{Y_{li}(X_{li}),X_{li},Z_{li}\}$. Under mild regularity conditions, we have the following consistency and asymptotic normality results.

Proposition 1 *Under the regularity Conditions (C1)‐(C5) in the Supporting Information and (A1)‐(A3) for the win function* 𝒲 *, as* n→∞ *, we have that* $\hat{\beta }\overset{p}{\to }\beta_{0}$ *and that*

$$\sqrt{n}(\hat{\beta }-\beta_{0})=-2{\left(\overset{L}{\underset{l=1}{\sum }}A_{l}\right)}^{-1}\overset{L}{\underset{l=1}{\sum }}\frac{\sqrt{n}}{n_{l}}\overset{n_{l}}{\underset{i=1}{\sum }}\kappa_{l}\left(O_{li}\right)+o_{p}(1),$$

*where*

$$A_{l}=-E\left(\frac{\exp\left\{\beta_{0}^{T}\left(Z_{li}-Z_{lj}\right)\right\}}{{\left[1+\exp\left\{\beta_{0}^{T}\left(Z_{li}-Z_{lj}\right)\right\}\right]}^{2}}{\left(Z_{li}-Z_{lj}\right)}^{⊗2}\int_{0}^{\tau }h_{l}\left(t;Z_{li},Z_{lj};\beta_{0}\right)R_{li,lj}(dt)\right)$$

*and* $\kappa_{l}\left(O_{li}\right)=E\left[\left(Z_{li}-Z_{lj}\right)\int_{0}^{\tau }h_{l}\left(t;Z_{li},Z_{lj};\beta_{0}\right)M_{li,lj}\left(dt|Z_{li},Z_{lj};\beta_{0}\right)|O_{li}\right]$.

The variance matrix of $\hat{\beta }$ can thus be estimated by

$${\hat{\sum }}_{1}=4{\left(\overset{L}{\underset{l=1}{\sum }}{\hat{A}}_{l}\right)}^{-1}\left\{\overset{L}{\underset{l=1}{\sum }}n_{l}^{-2}\overset{n_{l}}{\underset{i=1}{\sum }}{\hat{\kappa }}_{l}{\left(O_{li}\right)}^{⊗2}\right\}{\left(\overset{L}{\underset{l=1}{\sum }}{\hat{A}}_{l}\right)}^{-1},$$

where

A^l=−nl2−1∑i<jnl∑expβ^TZli−Zlj1+expβ^TZli−Zlj2Zli−Zlj⊗2∫0τh^lt;Zli,Zlj;β^Rli,lj(dt),

and ${\hat{\kappa }}_{l}\left(O_{li}\right)={\left(n_{l}-1\right)}^{-1}\sum_{j\neq i}^{n_{l}}\left(Z_{li}-Z_{lj}\right)\int_{0}^{\tau }{\hat{h}}_{l}(t;Z_{li},Z_{lj};\hat{\beta })M_{li,lj}(dt|Z_{li},Z_{lj};\hat{\beta }).$ The full derivation can be found in the Supporting Information.

### 3.2 Variance under diverging strata (L→∞)

When L is large, there may not be enough subjects in every stratum to justify an asymptotic expansion as in Proposition 1. But since L→∞, we can view the left hand side of (5) as the sum of L independent, albeit nonidentically distributed, stratum‐specific terms (each of which just happens to be a U‐statistic). To quantify the variance of this sum, we can use the usual Lindeberg‐Feller central limit theorem. The details are provided in the Supporting Information.

Proposition 2 Under regularity Conditions (C1)−(C3) and (C4*)−(C5*) in Supporting Information and (A1)−(A3) for the win function 𝒲 *, when* L→∞ *we have that*

$$S_{L}^{-1/2}\left(\overset{L}{\underset{l=1}{\sum }}A_{l}\right)(\hat{\beta }-\beta_{0})⇝{𝒩}_{p}(0,I),$$

*where*

$$S_{L}=\overset{L}{\underset{l=1}{\sum }}\mathrm{Var}\left\{{\left(\begin{array}{c} n_{l}\\ 2 \end{array}\right)}^{-1}\overset{n_{l}}{\underset{i<j}{\sum }}\sum \left(Z_{li}-Z_{lj}\right)\overset{\tau }{\underset{0}{\int }}h_{l}\left(t;Z_{li},Z_{lj};\beta_{0}\right)M_{li,lj}\left(dt|Z_{li},Z_{lj};\beta_{0}\right)\right\}.$$

The variance matrix of $\hat{\beta }$ can be estimated by

$${\hat{\sum }}_{2}={\left(\overset{L}{\underset{l=1}{\sum }}{\hat{A}}_{l}\right)}^{-1}{\hat{S}}_{L}{\left(\overset{L}{\underset{l=1}{\sum }}{\hat{A}}_{l}\right)}^{-1}$$

where

S^L=∑l=1Lnl2−1∑i<jnl∑Zli−Zlj∫0τh^lt;Zli,Zlj;β^Mli,ljdt|Zli,Zlj;β^⊗2.

Given α∈(0,1), the 100(1−α)% confidence interval for $\beta_{k}$ is $\left[{\hat{\beta }}_{k}-z_{1-\alpha /2}{\hat{\sigma }}_{k},\;\;{\hat{\beta }}_{k}+z_{1-\alpha /2}{\hat{\sigma }}_{k}\right]$, where $z_{1-\alpha /2}$ is the 100(1−α/2)% quantile of the standard normal distribution and ${\hat{\sigma }}_{k}$ is the square root of the kth diagonal element of either ${\hat{\sum }}_{1}$ or ${\hat{\sum }}_{2}$, depending the choice of the variance estimator. Then, the 100(1−α)% confidence interval for $\exp(\beta_{k})$, the win ratio with respect to the kth covariate, is $\left[\exp({\hat{\beta }}_{k}-z_{1-\alpha /2}{\hat{\sigma }}_{k}),\;\;\exp({\hat{\beta }}_{k}+z_{1-\alpha /2}{\hat{\sigma }}_{k})\right]$.

## 4 SIMULATION STUDIES

### 4.1 A small number of strata

In the first set of simulations, we assessed the estimation of regression parameters under a correctly specified SPW$\left({𝒲}_{P}\right)$. We set L=3 strata with stratum probabilities P(l=1)=0.2, P(l=2)=0.3, and P(l=3)=0.5. Let $Z={\left(Z_{1},Z_{2}\right)}^{T}$, where

$$Z_{1}|(Z_{2},l)∼\text{Bernoulli}\{\text{expit}(-0.2+0.5Z_{2}+0.2l)\}$$

with expit(x)=exp(x)/{1+exp(x)} and $Z_{2}∼N(0,1)$. Consider a composite endpoint consisting of survival time D and a nonfatal event time T. We generated their joint distribution under the stratified Gumbel‐Hougaard model

(7) $$P(D_{li}>s,T_{li}>t|Z_{li})=\exp\left\{-{\left[{\left\{\lambda_{Dl}\exp(-\beta^{T}Z)s\right\}}^{\kappa_{l}}+{\left\{\lambda_{Hl}\exp(-\beta^{T}Z)t\right\}}^{\kappa_{l}}\right]}^{1/\kappa_{l}}\right\}(l=1,2,3),$$

a special case of the stratified Lehmann model in (2). Under this model, the SPW assumption (1) is satisfied with $𝒲={𝒲}_{P}$ and regression parameter β. Let $\lambda_{Hl}=2+\mu_{H}(l-1)$, $\lambda_{Dl}=0.2+\mu_{D}(l-1)$, and $\kappa_{l}=2+(l-1)$ (resulting in between‐component Kendall's concordance 50%, 67%, and 75% for l=1,2, and 3, respectively^24^). The parameters $\mu_{H},\mu_{D}$, and $\mu_{A}$ control the between‐strata heterogeneity. We set $(\mu_{H},\mu_{D},\mu_{A})=$ (0.3,0.1,0.3), (0.5,0.2,0.5), and (1.0,0.5,0.5), with $\beta ={\left(\beta_{1},\beta_{2}\right)}^{T}={\left(-0.5,0.5\right)}^{T},{\left(0,0\right)}^{T},\;\text{and}\;{\left(0.5,-0.5\right)}^{T}$. Under this set‐up, the death and nonfatal event rates are about 35% and 65%, respectively.

We used the procedures described in Section 2.2 to fit SPW$\left({𝒲}_{P}\right)$, along with the variance estimator under finite strata (since L=3). We also fit the unstratified model as comparison. The results for the estimation of $\beta_{1}$ are summarized in Table 1. Each scenario is based on 2000 replicates. Because the relationship between $Z_{1}$ and (D,T) is confounded by the strata, the unstratified model produces considerable bias. The bias becomes greater as the between‐strata heterogeneity increases. By contrast, the stratified method stays largely unbiased in all scenarios. Its standard error estimator agrees well with the empirical standard error of ${\hat{\beta }}_{1}$. The 95% confidence interval has empirical coverage probabilities close to the nominal rate.

**TABLE 1:** Simulation results on the estimation of $\beta_{1}$

					PW				Stratified PW			
$\mu_{H}$	$\mu_{D}$	$\mu_{A}$	n	$\beta_{1}$	EST	SE	SEE	CP	EST	SE	SEE	CP
0.3	0.1	0.3	200	−0.5	−0.525	0.185	0.184	0.952	−0.502	0.190	0.189	0.952
				0.0	−0.023	0.180	0.177	0.951	−0.001	0.183	0.181	0.957
				0.5	0.467	0.181	0.187	0.955	0.493	0.185	0.192	0.960
			500	−0.5	−0.523	0.120	0.115	0.940	−0.502	0.121	0.117	0.942
				0.0	−0.024	0.112	0.111	0.940	−0.000	0.113	0.112	0.944
				0.5	0.481	0.116	0.117	0.949	0.507	0.117	0.119	0.960
			1000	−0.5	−0.520	0.081	0.081	0.943	−0.500	0.081	0.082	0.952
				0.0	−0.025	0.077	0.078	0.940	−0.001	0.078	0.078	0.958
				0.5	0.472	0.082	0.082	0.936	0.498	0.083	0.083	0.950
0.5	0.2	0.5	200	−0.5	−0.536	0.191	0.184	0.936	−0.510	0.193	0.189	0.947
				0.0	−0.036	0.179	0.177	0.944	0.004	0.183	0.181	0.949
				0.5	0.460	0.190	0.186	0.951	0.508	0.194	0.192	0.955
			500	−0.5	−0.528	0.119	0.115	0.934	−0.499	0.119	0.117	0.948
				0.0	−0.035	0.108	0.110	0.942	0.003	0.109	0.112	0.954
				0.5	0.454	0.118	0.116	0.926	0.500	0.118	0.118	0.944
			1000	−0.5	−0.525	0.081	0.081	0.936	−0.497	0.081	0.082	0.954
				0.0	−0.040	0.077	0.078	0.924	−0.001	0.077	0.078	0.960
				0.5	0.454	0.084	0.082	0.902	0.501	0.085	0.083	0.950
1.0	0.5	0.5	200	−0.5	−0.524	0.182	0.184	0.951	−0.501	0.186	0.188	0.953
				0.0	−0.073	0.178	0.176	0.929	−0.004	0.180	0.180	0.953
				0.5	0.400	0.183	0.184	0.919	0.501	0.187	0.190	0.952
			500	−0.5	−0.524	0.118	0.115	0.940	−0.498	0.120	0.116	0.941
				0.0	−0.069	0.112	0.110	0.910	0.000	0.112	0.111	0.940
				0.5	0.403	0.114	0.115	0.867	0.503	0.116	0.117	0.954
			1000	−0.5	−0.527	0.083	0.081	0.928	−0.500	0.083	0.082	0.951
				0.0	−0.064	0.077	0.077	0.870	0.004	0.078	0.078	0.951
				0.5	0.401	0.080	0.081	0.766	0.501	0.081	0.082	0.959

*Note*: EST and SE are the mean and standard error of the parameter estimator; SEE is the mean of the standard error estimator; CP is the coverage probability of the 95% confidence interval.

Next, we compared the stratified versus unstratified PW models in testing the hypothesis $H_{0}:\beta_{1}=0$. We used the same set‐up as in the previous simulations except that we fixed $\beta_{2}=-0.5$ and considered $\beta_{1}=$ 0, 0.2, and 0.5, corresponding to win ratio 1.00, 1.22, and 1.65, respectively. The empirical type I error (under win ratio 1.00) and power (under win ratios 1.22 and 1.65) are summarized in Table 2. Each scenario is based on 2,000 replicates. The type I error is correctly maintained under the stratified model, but is notably inflated under the unstratified model in the presence of high between‐strata heterogeneity. Throughout, the stratified model is considerably more powerful than the unstratified version, thanks to the efficiency gain by adjustment of strata.

**TABLE 2:** Empirical type I error and power by tests based on PW and Stratified PW model

				n=200		n=500	
$\mu_{H}$	$\mu_{D}$	$\mu_{A}$	Win ratio	PW	Stratified PW	PW	Stratified PW
0.3	0.1	0.3	1.00	0.050	0.046	0.058	0.051
			1.22	0.168	0.196	0.338	0.407
			1.65	0.719	0.742	0.990	0.991
0.5	0.2	0.5	1.00	0.054	0.049	0.066	0.042
			1.22	0.137	0.181	0.285	0.417
			1.65	0.690	0.752	0.974	0.988
1.0	0.5	0.5	1.00	0.063	0.051	0.101	0.052
			1.22	0.102	0.195	0.177	0.403
			1.65	0.591	0.768	0.947	0.993

### 4.2 A large number of strata

In this section, we assessed the performance of the estimation procedure and the variance estimator for SPW$\left({𝒲}_{P}\right)$ under a diverging number of strata. Let $Z_{1}∼\text{Bernoulli}\{\text{expit}(\gamma Z_{2})\}$ and $Z_{2}∼𝒩(0,1)$. For simplicity, we set a uniform stratum size $n_{l}=2$ or 4. For example, when the sample size n=200 and $n_{l}=2$, there will be L=100 strata with two subjects in each stratum (ie, a sample of matched pairs). For each l=1,…,L, we generated $\left(D_{li},T_{li}\right)$ under a frailty‐like version of (7) by letting $\lambda_{Hl}∼$ Gamma(shape = 2, rate = 2) and $\lambda_{Dl}∼0.5\;\times \;$Gamma(shape = 3, rate = 3), with a fixed $\kappa_{l}=2$. With $\beta ={\left(\beta_{1},\beta_{2}\right)}^{T}={\left(-0.5,0.5\right)}^{T},{\left(0,0\right)}^{T},\;\text{and}\;{\left(0.5,-0.5\right)}^{T}$, we simulated 2000 replicates for each scenario and estimated $\beta_{1}$, with standard error estimated by both approaches in Section 3. The results are summarized in Table 3. As before, the point estimator shows minimal bias. Meanwhile, its standard error is much more accurately estimated by the diverging‐strata approach than it is by the finite‐strata one (which tends to overestimate).

**TABLE 3:** Comparison between variance estimator under large and small strata

n	$n_{l}$	$\beta_{1}$	EST	SE	SEE (diverging strata)	SEE (finite strata)
200	2	−0.5	−0.496	0.357	0.344	0.508
		0.0	0.001	0.338	0.327	0.513
		0.5	0.498	0.361	0.361	0.537
	4	−0.5	−0.504	0.250	0.247	0.316
		0.0	−0.005	0.244	0.239	0.304
		0.5	0.493	0.270	0.260	0.333
500	2	−0.5	−0.488	0.220	0.213	0.315
		0.0	−0.006	0.202	0.203	0.318
		0.5	0.488	0.228	0.225	0.333
	4	−0.5	−0.492	0.159	0.157	0.199
		0.0	0.001	0.154	0.150	0.190
		0.5	0.493	0.172	0.164	0.209
1000	2	−0.5	−0.491	0.150	0.150	0.222
		0.0	−0.002	0.142	0.143	0.223
		0.5	0.485	0.162	0.157	0.233
	4	−0.5	−0.487	0.111	0.110	0.140
		0.0	0.001	0.109	0.106	0.134
		0.5	0.491	0.120	0.116	0.148

### 4.3 Comparison with Pocock's matched and unmatched win ratio

Finally, we compared the stratified PW model with the two‐sample win ratio of Pocock et al^5^ in hypothesis testing. Let $Z_{1}|Z_{2}∼\text{Bernoulli}\{\text{expit}(\gamma Z_{2})\}$, where $Z_{2}∼𝒩(0,1)$, and consider the comparison between $Z_{1}=1$ with $Z_{1}=0$. Let $Z_{3}∼𝒩(0,1)$ be another independent variable. We generated the composite outcomes using the Gumbel‐Hougaard model $P(D>s,T>t|Z)=\exp\left(-{\left[{\left\{\lambda_{D}\exp(-\beta^{T}Z)s\right\}}^{\kappa }+{\left\{\lambda_{H}\exp(-\beta^{T}Z)t\right\}}^{\kappa }\right]}^{1/\kappa }\right)$, where $Z={\left(Z_{1},Z_{2},Z_{3}\right)}^{T}$, $\lambda_{H}=2$, $\lambda_{D}=0.2$, and κ=2. We fixed $\beta_{2}=-0.5$, $\beta_{3}=1.2$, and set $\beta_{1}=0,0.2,0.5$, corresponding to win ratio 1.00, 1.22, and 1.65, respectively. To adjust for $Z_{2}$ as a potential confounder, we used logistic regression to fit the propensity score $P(Z_{1}=1|Z_{2})$, and matched one patient with $Z_{1}=1$ to one with $Z_{1}=0$.^25^ We then tested the effect of $Z_{1}$ under γ=0 (no confounding), 0.2 and 0.5 (confounding by $Z_{2}$) using Pocock's unmatched two‐sample win ratio, Pocock's matched two‐sample win ratio,^5^ and the Wald test on $\beta_{1}$ in the PW model stratified by the pairs with ${\left(Z_{1},Z_{3}\right)}^{T}$ as covariates. With n=500, the empirical type I error (under win ratio 1.00) and power (under win ratios 1.22 and 1.65) based on 2000 replicates are summarized in Table 4. Without confounding, all methods yield roughly correct type I error. In the presence of confounding, the type I error of the unmatched win ratio is severely inflated. The matched win ratio and stratified PW model maintain correct type I error, with the latter more powerful due to adjustment of $Z_{3}$.

**TABLE 4:** Empirical type I error and power by tests based on unmatched Pocock's win ratio, matched Pocock's win ratio and stratified PW model

Confounding	Win ratio	Two‐sample (unmatched)	Two‐sample (matched)	Stratified PW
None (γ=0.0)	1.00	0.057	0.055	0.053
	1.22	0.148	0.146	0.167
	1.65	0.761	0.542	0.669
Moderate (γ=0.2)	1.00	0.133	0.060	0.049
	1.22	0.062	0.120	0.148
	1.65	0.563	0.531	0.634
Strong (γ=0.5)	1.00	0.352	0.057	0.062
	1.22	0.076	0.115	0.119
	1.65	0.256	0.470	0.574

## 5 ANALYSIS OF THE ACCORD LIPID STUDY

The Action to Control Cardiovascular Risk in Diabetes (ACCORD) trial studied the effectiveness of intensive therapy (targeting a glycated hemoglobin level of less than 6%) versus standard therapy (targeting a level of 7% to 7.9%) in reducing the CV risk of 10 251 patients with type 2 diabetes.^26^ Under a double 2‐by‐2 factorial design, a subgroup of 5,518 patients were also enrolled in the ACCORD Lipid study, which compares the effects of simvastatin plus fenofibrate and simvastatin plus placebo on the patient's CV outcomes.^27^ The primary outcome was a composite of nonfatal myocardial infarction (MI), nonfatal stroke, and death of CV causes. Using time‐to‐first‐event methods, the primary analysis of the ACCORD Lipid trial showed a moderate beneficial effect of fenofibrate with a nonsignificant hazard ratio of 0.92. To illustrate our methods, we reanalyze certain subgroups of the study data using the (prioritized) composite endpoint of CV death and the first nonfatal event (MI or stroke).

### 5.1 Sex‐stratified analysis

We first consider a subgroup of patients with CV disease history and a low level of baseline high‐density lipoprotein cholesterol (≤ 40 mg/dl). The original analysis of the ACCORD Lipid trial suggests that this subgroup may benefit more from the fenofibrate treatment than the average patient does.^27^ As seen from Table 5, baseline features are similar across the two groups. The overall median follow‐up time was 4.7 years with a first‐event rate of 3.9% and a CV death rate of 1.4%.

**TABLE 5:** Baseline features of the ACCORD Lipid study subset in Section 5.1

Variable	Fenofibrate (N = 715)	Placebo (N = 710)	Overall (N = 1425)
Age (year)	62.5 (57.5‐68.2)	62.6 (57.6‐68.0)	62.6 (57.5‐68.1)
Female (no.) (%)	108 (15.7%)	114 (15.9%)	222 (15.6%)
Intensive therapy (no.) (%)	358 (50.4%)	370 (51.7%)	728 (51.1%)
Race (no.) (%)			
White	513 (72.3%)	514 (71.9%)	1027 (72.1%)
Black	73 (10.3%)	84 (11.7%)	157 (11.0%)
Hispanic	43 (6.1%)	48 (6.7%)	91 (6.4%)
Other	81 (11.4%)	69 (9.7%)	150 (10.5%)
Glycated hemoglobin (%)	8.2 (7.7‐8.8)	8.1 (7.6‐8.8)	8.2 (7.6‐8.8)
Plasma cholesterol (mg/dl)			
Total	158 (140‐185)	160 (140‐187)	160 (140‐186)
Low‐density lipoprotein	88 (72‐107)	90 (73‐110)	89 (72‐108)
High‐density lipoprotein	33 (30‐37)	34 (30‐37)	34 (30‐37)
Plasma triglyceride (mg/dl)	182 (129‐248)	172 (120‐244)	178 (125‐246)
First‐event rate (${\text{year}}^{-1}$)	0.037	0.042	0.039
CV death rate (${\text{year}}^{-1}$)	0.013	0.016	0.014

*Note*: Quantitative variables are summarized by median (inter‐quartile range) and categorical variables by N(%).

**FIGURE 1:**
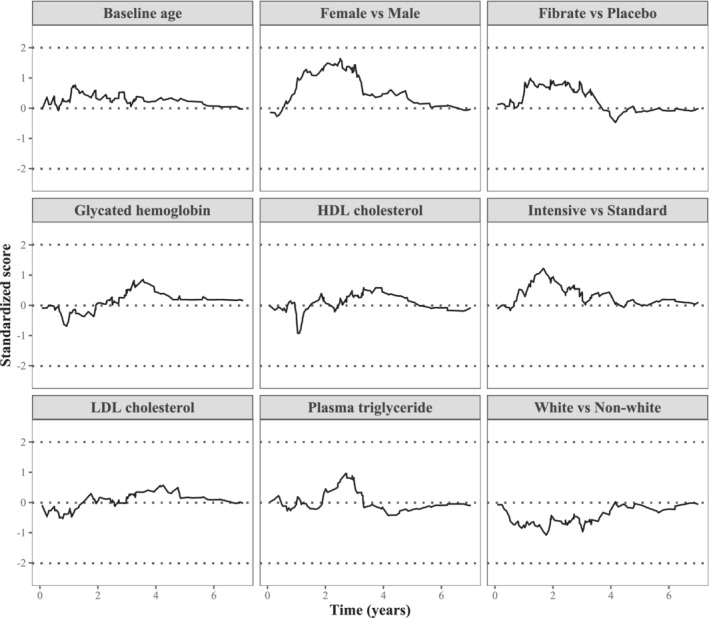
Standardized score processes for all covariates in the ACCORD Lipid study data in Section 5.1

We first fit an unstratified PW model under ${𝒲}_{P}$ with fenofibrate vs placebo, intensive vs standard therapy, patient age, sex, race, glycated hemoglobin, HDL cholesterol, LDL cholesterol and plasma triglyceride as covariates. Figure 1 shows the standardized score processes for checking the proportionality assumption on each covariate.^4^ Per the rule of thumb, most curves are well behaved, fluctuating randomly around zero between −2 and 2. The only variable that shows a somewhat concerning trend is sex, which we can address by stratifying the model on it. Since sex only has two levels, we use the variance estimator for finite strata. The standardized score processes for the resulting SPW$\left({𝒲}_{P}\right)$ appear mostly benign (see Figure S1 in the Supporting Information). The inference results are summarized in Table 6. Within each sex and adjusting for other predictors, patients treated by fenofibrate are 17% more likely to have a favorable composite outcome, that is, with delayed CV death and the first nonfatal MI or stroke, compared to those receiving placebo. This effect, however, is nonsignificant at level 0.05. Meanwhile, younger age and higher HDL cholesterol are significantly associated with a more favorable outcome.

**TABLE 6:** Sex‐stratified PW regression analysis of the ACCORD Lipid study

	Sex‐stratified PW		
Variable	Win ratio	95% confidence interval	P‐value
Fibrate vs Placebo	1.17	(0.90, 1.51)	0.243
Intensive vs Standard	0.87	(0.67, 1.12)	0.273
Baseline age (years)	0.97	(0.96, 0.99)	0.005
White vs Nonwhite	0.86	(0.63, 1.18)	0.360
Glycated hemoglobin (%)	0.93	(0.82, 1.06)	0.308
Plasma triglyceride (mg/dl)	1.00	(1.00, 1.00)	0.978
LDL cholesterol (mg/dl)	1.00	(1.00, 1.01)	0.797
HDL cholesterol (mg/dl)	1.04	(1.01, 1.07)	0.009

### 5.2 Age‐stratified analysis

Next, we consider another subgroup of patients with a high level of baseline triglyceride level (≥ 204 mg/dl) and a low level of baseline high‐density lipoprotein cholesterol (≤ 34 mg/dl). This group also responded particularly well according to the original analysis. Some key baseline features were summarized in Table S1 of the Supporting Information.

Because age is a common source of heterogeneity to many clinical outcomes and it is often difficult to model its effects correctly, we fit a PW model stratified by age and use the remaining variables as covariates. Specifically, we cut age into 56 intervals by [0, 50), [50, 50.5), [50.5, 51), …, [76.5, 77) and [77, ∞), with about 10 patients per interval. Given the large number of strata and their relatively small sizes, we used the variance estimator designed for diverging strata to make inferences. The standardized score processes plotted in Figure S2 in the Supporting Information show no obvious signs of nonproportionality. Table 7 summarizes the results of this age‐stratified PW model. In each age group, patients under fenofibrate treatment are 56% more likely to have a favorable composite outcome by delaying CV death and the first nonfatal MI or stroke as compared to those under placebo (P‐value 0.009). In addition, absence of CV disease history and lower levels of glycated hemoglobin are significantly associated with a favorable outcome.

**TABLE 7:** Age‐stratified PW regression analysis of the ACCORD lipid study

	Age‐stratified PW		
Variable	Win ratio	95% confidence interval	P‐value
Fibrate vs Placebo	1.56	(1.12, 2.19)	0.009
Intensive vs Standard	1.04	(0.72, 1.49)	0.840
Female vs Male	1.10	(0.63, 1.90)	0.739
CVD Event History	0.44	(0.30, 0.64)	<0.001
White vs Nonwhite	1.06	(0.68, 1.63)	0.803
Glycated hemoglobin (%)	0.81	(0.67, 0.98)	0.030
LDL cholesterol (mg/dl)	1.00	(0.99, 1.01)	0.949
HDL cholesterol (mg/dl)	0.98	(0.94, 1.03)	0.472

## 6 DISCUSSIONS

We have studied the specification, estimation, and inference of stratified PW models. Stratification allows us to dispense with the proportionality assumption in accounting for patient heterogeneity, thereby providing a flexible way to adjust for confounding and to increase efficiency. From a substantive point of view, the within‐stratum comparisons may also appeal to practitioners who want to restrict analysis to patients under the same exposure or of the same disease type. Besides the standard case with a small number of strata, our inference framework also allows the number of strata to diverge with the sample size. This effectively extends the matched win ratio of Pocock et al^5^ from two‐sample comparison to regression, enabling researchers to fit PW models to matched pairs that frequently arise in observational studies. It also allows them to stratify on a continuous variable by cutting it into many groups (as we did in Section 5.2). Nevertheless, clinically meaningful cutoffs may prove elusive, and one must balance statistical versus substantive considerations in choosing the thresholds.

The proposed U‐statistics estimating equations allow user‐specified, stratum‐specific, and time‐dependent weight functions. The choice of weights will not affect the interpretation of the regression parameter, but will determine the statistical efficiency of the resulting estimator. The simple choice of $n_{l}/n$ may not be the most efficient one, especially in the presence of strong between‐strata heterogeneity. It remains an open problem to find optimal weights in general.

The examples in Section 5 are analyzed by Pocock's win function ${𝒲}_{P}$, where nonfatal events are compared only up to time to the first occurrence. When those events are numerous, one may prefer a win function that make fuller use of the data, both for statistical efficiency and clinical interpretation. To illustrate, we include in the Supporting Information another data example with all‐cause death and repeated hospitalizations analyzed by the recurrent‐event win function described in Section 2.1. Since different win functions entail different proportionality assumptions, one can use the score processes introduced in Section 2.3 to aid in their selection.

The two types of variance estimators are developed based on two clean‐cut asymptotic settings—either the size of each stratum or the total number of strata goes to infinity. In practice, it may happen that neither the size nor the number are large enough to allow the corresponding asymptotics to kick in. It will be of interest to develop a more general approach that guarantees satisfactory performance as long as the total sample size is large. This would allow the analyst to avoid making a (sometimes arbitrary) choice between the two types of variance estimators.

We have considered a standard stratification scheme in which the strata are pre‐specified with constant covariate effects across. This may lose efficiency if the user‐defined strata are misaligned with the underlying heterogeneity structure,^28^ and may even incur bias if there are non‐negligible interactions between the strata and covariates. Recently, Ito and Sugasawa^29^ sought to determine strata empirically and to allow for stratum‐specific regression parameters in generalized estimating equations for longitudinal data. The SPW models may be improved along such lines in the future.

## Supporting information




**Appendix S1** Supplementary Material

## Data Availability

The data that support the findings in this article are available on request from the corresponding author. The data are not publicly available due to privacy or ethical restrictions.
