# Supplementary material for: Stratified proportional win‐fractions regression analysis
Source: Stat Med. 2022 Sep 14;41(26):5305–18. doi: 10.1002/sim.9570 (PMC9826339; doi:10.1002/sim.9570)
Supplement: Supplementary file 1 — Appendix S1 Supplementary Material [file SIM-41--s001.pdf]

Received: Added at production | Revised: Added at production | Accepted: Added at production
DOI: xxx/xxxx

STATISTICS IN MEDICINE

Supporting Information for "Stratified proportional win-fractions regression analysis"

Tuo Wang | Lu Mao*

Department of Biostatistics and Medical Informatics, University of Wisconsin-Madison, Wisconsin, USA

Correspondence
*Lu Mao, Email: lmao@biostat.wisc.edu

Summary

This web appendix contains technical details, proofs and additional tables and figures referenced throughout the main text.

S1 | TECHNICAL RESULTS AND PROOFS

S1.1 | Win function on recurrent events

For notational simplicity, consider only one counting process $N(t)$ for recurrent nonfatal events. Let $T_1 < T_2 < \dots$ denote the ordered times to the recurrent events counted by $N(t)$. Following Mao et al. (2022)[1], we can first compare on survival, and then the cumulative frequency of recurrent events, with ties further broken by time to the last occurrence. This leads to

$$\mathcal{W}_R(\boldsymbol{Y}_{li}, \boldsymbol{Y}_{lj})(t) = I(D_{lj} < D_{li} \wedge t) + I\{D_{lj} \wedge D_{li} > t, N_{li}(t) < N_{lj}(t)\} \\ + I\{D_{lj} \wedge D_{li} > t, N_{li}(t) = N_{lj}(t) = k > 1, T_{klj} < T_{kli} \wedge t\},$$

where T_{kli} and T_{kli} are T_k on the ith and jth subject in the lth stratum, respectively. Extending §2.4 of Mao et al.[1] slightly, we find that SPW($\mathcal{W}_R$) holds with regression parameter $\boldsymbol{\beta}$ if

$$P(D_{li} > s, T_{1li} > t_1, T_{2li} > t_2, \dots \mid \boldsymbol{Z}_{li}) = H_l(s, t_1, t_2, \dots)^{\exp(-\boldsymbol{\beta}^T \boldsymbol{Z}_{li})}, \tag{S1}$$

where $H_l(s, t_1, t_2, \dots)$ is a nonparametric baseline joint survival function for $(D, T_1, T_2, \dots)$. Like the counterexample offered in Mao and Wang (2021)[2], model (S1) is sufficient but far from necessary for SPW($\mathcal{W}_R$).

S1.2 | Efficiency of weights

Here we give a heuristic argument about the efficiency of weights. Consider only time-constant weights but allow weights to vary across strata, i.e. $\widehat{h}_l = c_l$ for $l = 1, \dots, L$, where c_l's are some constants. Without loss of generality, assume $\sum_{l=1}^L c_l = 1$ and for simplicity, assume Z is one dimensional. Then for each U-statistic estimating function in stratum l, we have

$$\binom{n_l}{2}^{-1} \sum_{i<j}^{n_l} \sum \left(Z_{li} - Z_{lj}\right) \int_0^\tau \widehat{h}_l\left(t; Z_{li}, Z_{lj}; \boldsymbol{\beta}\right) M_{li,lj}\left(\mathrm{d}t \mid Z_{li}, Z_{lj}; \boldsymbol{\beta}\right) = c_l s_l,$$

where $s_l = \binom{n_l}{2}^{-1} \sum_{i<j}^{n_l} \sum \left(Z_{li} - Z_{lj}\right) M_{li,lj}\left(\tau \mid Z_{li}, Z_{lj}; \boldsymbol{\beta}\right)$. The variance of $\bar{s} = \sum_{l=1}^L c_l s_l$ is $\mathrm{Var}(\bar{s}) = \sum_{l=1}^L c_l^2 \mathrm{Var}(s_l)$. We want to minimize $f(c_1, \dots, c_L) = \sum_{l=1}^L c_l^2 \mathrm{Var}(s_l)$ subject to $\sum_{l=1}^L c_l = 1$. Construct the Lagrangian function $g(c_1, \dots, c_L) = f(c_1, \dots, c_L) + \lambda(1 - \sum_{l=1}^L c_l)$, take derivatives with respect to c_l's and λ, and assign the derivatives to zero, we can get

$$\lambda = \frac{1}{\sum_{l=1}^L \frac{1}{\mathrm{Var}(s_l)}}, \text{ and } c_l = \frac{1}{\left(\sum_{l'=1}^L \frac{1}{\mathrm{Var}(s_{l'})}\right) \mathrm{Var}(s_l)}.$$

Thus, $\text{Var}(\bar{s})$ is minimized when $c_l \propto \frac{1}{\text{Var}(s_l)}$. If the $\left(Z_{li} - Z_{lj}\right) M_{li,lj}\left(\tau \mid Z_{li}, Z_{lj}; \beta\right)$ are identically distributed for $l = 1, \dots, L$, then $\text{Var}(s_l) \propto \frac{1}{n_l}$ so that $c_l \propto n_l$.

S1.3 | Proof of Proposition 1

The regularity conditions for Theorem 1 are:

- (C1) The win indicator $\mathcal{W}(\boldsymbol{Y}_{li}, \boldsymbol{Y}_{li})(\cdot)$ has uniformly bounded total variation on $[0, \tau]$, i.e., $\exists M$ s.t.
$$\int_0^\tau |\mathcal{W}(\boldsymbol{Y}_{li}, \boldsymbol{Y}_{li})(t)| dt \le M < \infty.$$
- (C2) The support of $\boldsymbol{Z}$, denoted by $\mathcal{Z}$, is a bounded subset of $\mathbb{R}^p$, i.e. $\exists M$ s.t. $||\boldsymbol{Z}|| \le M, \forall \boldsymbol{Z} \in \mathcal{Z}$, where $||\cdot||$ denotes the Euclidean norm.
- (C3) $\boldsymbol{\beta}_0$, the true value of $\boldsymbol{\beta}$, lies in the interior of a compact subset $\mathcal{B}$ of $\mathbb{R}^p$
- (C4) The censoring time is continuously distributed on $[0, \tau]$ with $P(C \ge \tau \mid \boldsymbol{Z}) \ge c_0 > 0$ almost surely for some c_0.
- (C5) The weight function satisfies
$$||\widehat{h}_l - h_l||_\infty \equiv \sup_{(z,z^*)\in\mathcal{Z}^{\otimes 2}, \beta\in\mathcal{B}, t\in[0,\tau]} |\widehat{h}_l - h_l|(t; z, z^*; \boldsymbol{\beta}) \xrightarrow{p} 0,$$
for a fixed symmetric function $h_l(t; \cdot, \cdot; \boldsymbol{\beta})$. The limit function $h_l(t; \cdot, \cdot; \boldsymbol{\beta})$ is uniformly bounded with uniformly bounded total variation, is uniformly continuous in $\boldsymbol{\beta}$, and satisfies
$$\inf_{\beta\in\mathcal{B}} \int_0^\tau h_l\left(t; \boldsymbol{Z}_{li}, \boldsymbol{Z}_{lj}; \boldsymbol{\beta}\right) E\left\{R_{li,lj}(\mathrm{d}t) \mid \boldsymbol{Z}_{li}, \boldsymbol{Z}_{lj}\right\} \ge \epsilon > 0 \text{ almost surely for some } \epsilon.$$

Let $\widehat{\boldsymbol{\beta}}$ be the estimator obtained from solving (5) of the main text. We first prove consistency. Define

$$\begin{aligned}\mathbb{M}_n(\boldsymbol{\beta}) &= \sum_{l=1}^L \binom{n_l}{2}^{-1} \sum_{i<j}^n \sum \int_0^\tau \hat{h}_l\left(t; \boldsymbol{Z}_{li}, \boldsymbol{Z}_{lj}; \widehat{\boldsymbol{\beta}}\right) \left[\boldsymbol{\beta}^{\mathrm{T}}\left(\boldsymbol{Z}_{li} - \boldsymbol{Z}_{lj}\right) \delta_{li,lj}(\mathrm{d}t) - \log\left\{1 + e^{\boldsymbol{\beta}^{\mathrm{T}}(Z_{li} - Z_{lj})}\right\} R_{li,lj}(\mathrm{d}t)\right] \\ &=: \sum_{l=1}^L \mathbb{M}_{l,n_l}(\boldsymbol{\beta}).\end{aligned}$$

Therefore, $\frac{\partial \mathbb{M}_n(\boldsymbol{\beta})}{\partial \boldsymbol{\beta}}|_{\boldsymbol{\beta}=\widehat{\boldsymbol{\beta}}} = 0$. Since

$$\frac{\partial^2}{\partial \boldsymbol{\beta}^{\otimes 2}} \mathbb{M}_n(\boldsymbol{\beta}) = -\sum_{l=1}^L \binom{n_l}{2}^{-1} \sum_{i<j}^n \sum \left(\frac{e^{\boldsymbol{\beta}^{\mathrm{T}}(Z_{li} - Z_{lj})}}{\left[1 + e^{\boldsymbol{\beta}^{\mathrm{T}}(Z_{li} - Z_{lj})}\right]^2} \left(\boldsymbol{Z}_{li} - \boldsymbol{Z}_{lj}\right)^{\otimes 2} \right) \int_0^\tau \hat{h}_l\left(t; \boldsymbol{Z}_{li}, \boldsymbol{Z}_{lj}; \widehat{\boldsymbol{\beta}}\right) R_{li,lj}(\mathrm{d}t),$$

which is globally negative definite under (C2), (C3) and (C5). Thus, $\mathbb{M}_n(\boldsymbol{\beta})$ is globally concave and $\widehat{\boldsymbol{\beta}} = \arg\max_{\boldsymbol{\beta}} \mathbb{M}_n(\boldsymbol{\beta})$. Because the parameter space $\mathcal{B}$ is compact, we may assume without loss of generality that $\widehat{\boldsymbol{\beta}} \xrightarrow{p} \boldsymbol{\beta}^*$ for some $\boldsymbol{\beta}^* \in \mathcal{B}$. From the supporting information S.1.4 of Mao and Wang (2021), under (C1)-(C5), $\mathbb{M}_{l,n_l}(\boldsymbol{\beta})$ converges uniformly in $\boldsymbol{\beta}$ to

$$\mathcal{M}_l(\boldsymbol{\beta}) = E\left(\int_0^\tau h_l\left(t; \boldsymbol{Z}_{li}, \boldsymbol{Z}_{lj}; \boldsymbol{\beta}^*\right) \left[\boldsymbol{\beta}^{\mathrm{T}}\left(\boldsymbol{Z}_{li} - \boldsymbol{Z}_{lj}\right) \delta_{li,lj}(\mathrm{d}t) - \log\left\{1 + e^{\boldsymbol{\beta}^{\mathrm{T}}(Z_{li} - Z_{lj})}\right\} R_{li,lj}(\mathrm{d}t)\right]\right).$$

Thus, $\mathbb{M}_n(\boldsymbol{\beta}) = \sum_{l=1}^L \mathbb{M}_{l,n_l}(\boldsymbol{\beta})$ converge uniformly in $\boldsymbol{\beta}$ to $\mathcal{M}(\boldsymbol{\beta}) = \sum_{l=1}^L \mathcal{M}_l(\boldsymbol{\beta})$. We can show similarly that $\mathcal{M}(\boldsymbol{\beta})$ is globally concave so that it has a unique maximizer $\boldsymbol{\beta}_0$. Thus, by Theorem 5.7 of van der Varrt (1998), $\widehat{\boldsymbol{\beta}} \xrightarrow{p} \boldsymbol{\beta}_0$.[3]

Now we prove asymptotic linearity. Write $\boldsymbol{O}_{li} = \{\boldsymbol{Y}_{li}(X_{li}), X_{li}, \boldsymbol{Z}_{li}\}$ and define $\boldsymbol{f}_{\beta,h_l(\beta^*),l}\left(\boldsymbol{O}_{li}, \boldsymbol{O}_{lj}\right) = \int_0^\tau \left(\boldsymbol{Z}_{li} - \boldsymbol{Z}_{lj}\right) h_l\left(t; \boldsymbol{Z}_{li}, \boldsymbol{Z}_{lj}; \boldsymbol{\beta}^*\right) M_{li,lj}\left(\mathrm{d}t \mid \boldsymbol{Z}_{li}, \boldsymbol{Z}_{lj}; \boldsymbol{\beta}\right)$, where $h_l(\boldsymbol{\beta}) = h_l\left(t; \boldsymbol{Z}_{li}, \boldsymbol{Z}_{lj}; \boldsymbol{\beta}\right)$, and define

$$\mathbb{U}_{n_l}\boldsymbol{f}_{\beta,h_l(\beta^*),l} = \binom{n_l}{2}^{-1} \sum_{i<j}^{n}\sum \int_0^\tau \left(\boldsymbol{Z}_{li} - \boldsymbol{Z}_{lj}\right) h_l\left(t; \boldsymbol{Z}_{li}, \boldsymbol{Z}_{lj}; \boldsymbol{\beta}^*\right) M_{li,lj}\left(\mathrm{d}t \mid \boldsymbol{Z}_{li}, \boldsymbol{Z}_{lj}; \boldsymbol{\beta}\right).$$

Thus, $\widehat{\boldsymbol{\beta}}$ satisfies

$$\sum_{l=1}^{L} \mathbb{U}_{n_l}\boldsymbol{f}_{\widehat{\beta},\widehat{h}_l(\widehat{\beta}),l} = 0,$$

and $\boldsymbol{\beta}_0$ satisfies

$$\sum_{l=1}^{L} P \times P\left(\boldsymbol{f}_{\beta_0,h_l(\beta),l}\right) = 0, \quad \forall h_l, \forall \boldsymbol{\beta} \in \mathcal{B}.$$

From supporting information S.1.4 of Mao and Wang (2021), we have

$$\sqrt{n_l}\left(\mathbb{U}_{n_l} - P \times P\right) \boldsymbol{f}_{\widehat{\beta},\widehat{h}_l(\widehat{\beta}),l} = \sqrt{n_l}\left(\mathbb{U}_{n_l} - P \times P\right) \boldsymbol{f}_{\beta_0,h_l(\beta_0),l} + o_p(1).$$ [2]

Rearrange and sum across strata,

$$\sqrt{n}\sum_{l=1}^{L} P \times P\left(\boldsymbol{f}_{\widehat{\beta},\widehat{h}_l(\widehat{\beta}),l} - \boldsymbol{f}_{\beta_0,h_l(\beta_0),l}\right) = -\sqrt{n}\sum_{l=1}^{L} \mathbb{U}_{n_l}\boldsymbol{f}_{\beta_0,h_l(\beta_0),l} + \sum_{l=1}^{L}\frac{\sqrt{n}}{\sqrt{n_l}} o_p(1). \tag{S2}$$

Using Taylor expansion, the left hand side of (S2) is

$$\left(\sum_{l=1}^{L}\boldsymbol{A}_l\right)\sqrt{n}(\widehat{\boldsymbol{\beta}} - \boldsymbol{\beta}_0) + o_p(\sqrt{n}||\widehat{\boldsymbol{\beta}} - \boldsymbol{\beta}_0||),$$

where

$$\boldsymbol{A}_l = -E\left(\frac{\exp\left\{\boldsymbol{\beta}_0^{\mathrm{T}}\left(\boldsymbol{Z}_{li} - \boldsymbol{Z}_{lj}\right)\right\}}{\left[1 + \exp\left\{\boldsymbol{\beta}_0^{\mathrm{T}}\left(\boldsymbol{Z}_{li} - \boldsymbol{Z}_{lj}\right)\right\}\right]^2}\left(\boldsymbol{Z}_{li} - \boldsymbol{Z}_{lj}\right)^{\otimes 2}\int_0^\tau h_l\left(t; \boldsymbol{Z}_{li}, \boldsymbol{Z}_{lj}; \boldsymbol{\beta}_0\right) R_{li,lj}(\mathrm{d}t)\right)$$

Using Hoeffding decomposition, the right hand side of (S2) is

$$\sqrt{n}\sum_{l=1}^{L}\frac{1}{n_l}\sum_{i=1}^{n_l} -2\kappa_l\left(\boldsymbol{O}_{li}\right) + o_p(1),$$

where $\boldsymbol{\kappa}_l\left(\boldsymbol{O}_{li}\right) = E\left[\left(\boldsymbol{Z}_{li} - \boldsymbol{Z}_{lj}\right)\int_0^\tau h_l\left(t; \boldsymbol{Z}_{li}, \boldsymbol{Z}_{lj}; \boldsymbol{\beta}\right) M_{li,lj}\left(\mathrm{d}t \mid \boldsymbol{Z}_{li}, \boldsymbol{Z}_{lj}; \boldsymbol{\beta}\right) \mid \boldsymbol{O}_{li}\right]$.[4]

S1.4 | Proof of Proposition 2

Denote

$$\boldsymbol{F}_{\beta,h_l(\beta^*),l} = \binom{n_l}{2}^{-1}\sum_{i<j}^{n_l}\sum\left(\boldsymbol{Z}_{li} - \boldsymbol{Z}_{lj}\right)\int_0^\tau h_l\left(t; \boldsymbol{Z}_{li}, \boldsymbol{Z}_{lj}; \boldsymbol{\beta}^*\right) M_{li,lj}\left(\mathrm{d}t \mid \boldsymbol{Z}_{li}, \boldsymbol{Z}_{lj}; \boldsymbol{\beta}\right).$$

Thus, we have

$$\sum_{l=1}^{L}\boldsymbol{F}_{\widehat{\beta},\widehat{h}_l(\widehat{\beta}),l} = 0,$$

$$\mathrm{E}\boldsymbol{F}_{\beta_0,h_l(\beta),l} = 0, \ \forall h_l \text{ and } \boldsymbol{\beta} \in \mathcal{B}.$$

Denote $\boldsymbol{Q}_l = \mathrm{Var}\boldsymbol{F}_{\beta_0,\widehat{h}_l(\widehat{\beta}),l} = \mathrm{E}\left(\boldsymbol{F}_{\beta_0,\widehat{h}(\widehat{\beta}),l}\right)^{\otimes 2}$. The regularity conditions for Theorem 2 are (C1)-(C3) and

- (C4*) The weight functions $\widehat{h}_l$ are uniformly bounded.
- (C5*) The $\boldsymbol{Q}_l$ are positive definite and satisfy

$$\lim_{L\to\infty}\frac{1}{L}\sum_{l=1}^{L}\boldsymbol{Q}_l = \boldsymbol{Q}.$$

Under (C1)-(C3) and (C4*), $\{\boldsymbol{F}_{\beta_0,\hat{h}_l(\hat{\beta}),l}, l = 1, \dots, L\}$ is uniformly bounded, which further implies that

$$\frac{1}{L}\sum_{l=1}^{L} E||\boldsymbol{F}_{\beta_0,\hat{h}(\hat{\beta}),l}||^2 I\left\{||\boldsymbol{F}_{\beta_0,\hat{h}(\hat{\beta}),l}|| > \epsilon\sqrt{L}\right\} \to 0, \forall\epsilon > 0 \text{ as } L \to \infty.$$

Together with (C5*), by using the Linderberg-Feller central limit theorem, we have

$$\frac{1}{\sqrt{L}}\sum_{l=1}^{L} \boldsymbol{F}_{\beta_0\hat{h}_l(\hat{\beta}),l} \rightsquigarrow \mathcal{N}_p(0, \boldsymbol{Q}).^3$$

Because $\hat{\boldsymbol{\beta}}$ is the root of the estimating equations, $\hat{\boldsymbol{\beta}} \xrightarrow{p} \boldsymbol{\beta}_0$ and using Taylor expansion, we have

$$\begin{aligned}\sum_{l=1}^{L} \boldsymbol{F}_{\beta_0,\hat{h}_l(\hat{\beta}),l} &= -\sum_{l=1}^{L}\left(\boldsymbol{F}_{\hat{\beta},\hat{h}_l(\hat{\beta}),l} - \boldsymbol{F}_{\beta_0,\hat{h}_l(\hat{\beta}),l}\right)\\ &= \left(\sum_{l=1}^{L}\boldsymbol{A}_l\right)\left(\hat{\boldsymbol{\beta}} - \boldsymbol{\beta}_0\right) + o_p(||\hat{\boldsymbol{\beta}} - \boldsymbol{\beta}_0||)\end{aligned}$$

Thus,

$$\frac{1}{\sqrt{L}}\left(\sum_{l=1}^{L}\boldsymbol{A}_l\right)\left(\hat{\boldsymbol{\beta}} - \boldsymbol{\beta}_0\right) \rightsquigarrow \mathcal{N}_p(0, \boldsymbol{Q}).$$

Then variance of $\hat{\boldsymbol{\beta}}$ can be estimated by diagonal elements of

$$\left(\sum_{l=1}^{L}\hat{\boldsymbol{A}}_l\right)^{-1}\hat{\boldsymbol{S}}_L\left(\sum_{l=1}^{L}\hat{\boldsymbol{A}}_l\right)^{-1}$$

where

$$\hat{\boldsymbol{S}}_L = \sum_{l=1}^{L}\left\{\binom{n_l}{2}^{-1}\sum_{i<j}^{n_l}\sum\left(\boldsymbol{Z}_{li} - \boldsymbol{Z}_{lj}\right)\int_0^{\tau}\hat{h}_l\left(t; \boldsymbol{Z}_{li}, \boldsymbol{Z}_{lj}; \hat{\boldsymbol{\beta}}\right)M_{li,lj}\left(\mathrm{d}t \mid \boldsymbol{Z}_{li}, \boldsymbol{Z}_{lj}; \hat{\boldsymbol{\beta}}\right)\right\}^{\otimes 2}$$

S2 | ADDITIONAL FIGURES AND TABLES FOR THE ACCORD STUDY DATASET

Figures S1 and S2 show the standardized score processes for the sex- and age-stratified analyses in Sections 5.1 and 5.2, respectively. Table S1 summarizes patient characteristics of the study cohort in Section 5.2.

S3 | ANALYSIS OF THE HF-ACTION STUDY BY SPW($\mathcal{W}_R$)

We conducted additional analyses on a cardiovascular trial to illustrate the applications of SPW models on recurrent events, using the win function $\mathcal{W}_R$ described in Section S1.1. The Heart Failure: A Controlled Trial Investigating Outcomes of Exercise Training (HF-ACTION) trial was a randomized controlled clinical trial conducted on a cohort of over 2,000 patients to evaluate the efficacy and safety of exercise training among patients with heart failure.[5] The primary endpoint was a composite of all-cause mortality and all-cause hospitalizations. The primary analysis showed a moderate beneficial effect of exercise training in reducing the risk of the first composite event compared to usual care alone with a nonsignificant hazard ratio of 0.93 (P-value = 0.13).

We reanalyzed the HF-ACTION study by modeling all-cause death and repeated hospitalizations using SPW($\mathcal{W}_R$). The study cohort consists of 2,130 patients, with 1,060 randomized to exercise training and 1,070 to usual care. The mortality rate in the training arm is about 15.8%, with an average of 2.0 hospitalizations per patient; the mortality rate in the usual care arm is about 17.1%, with an average of 2.0 hospitalizations per patient. We included the treatment indicator, sex, age, and body mass index (BMI) in the SPW model stratified by heart failure etiology, i.e., nonischemic or ischemic. Table S2 summarizes the estimated win ratio and confidence intervals, constructed using the variance estimator under finite strata. Within each etiology group and adjusting for other predictors, patients going through exercise training are 6% more likely to have a favorable composite outcome

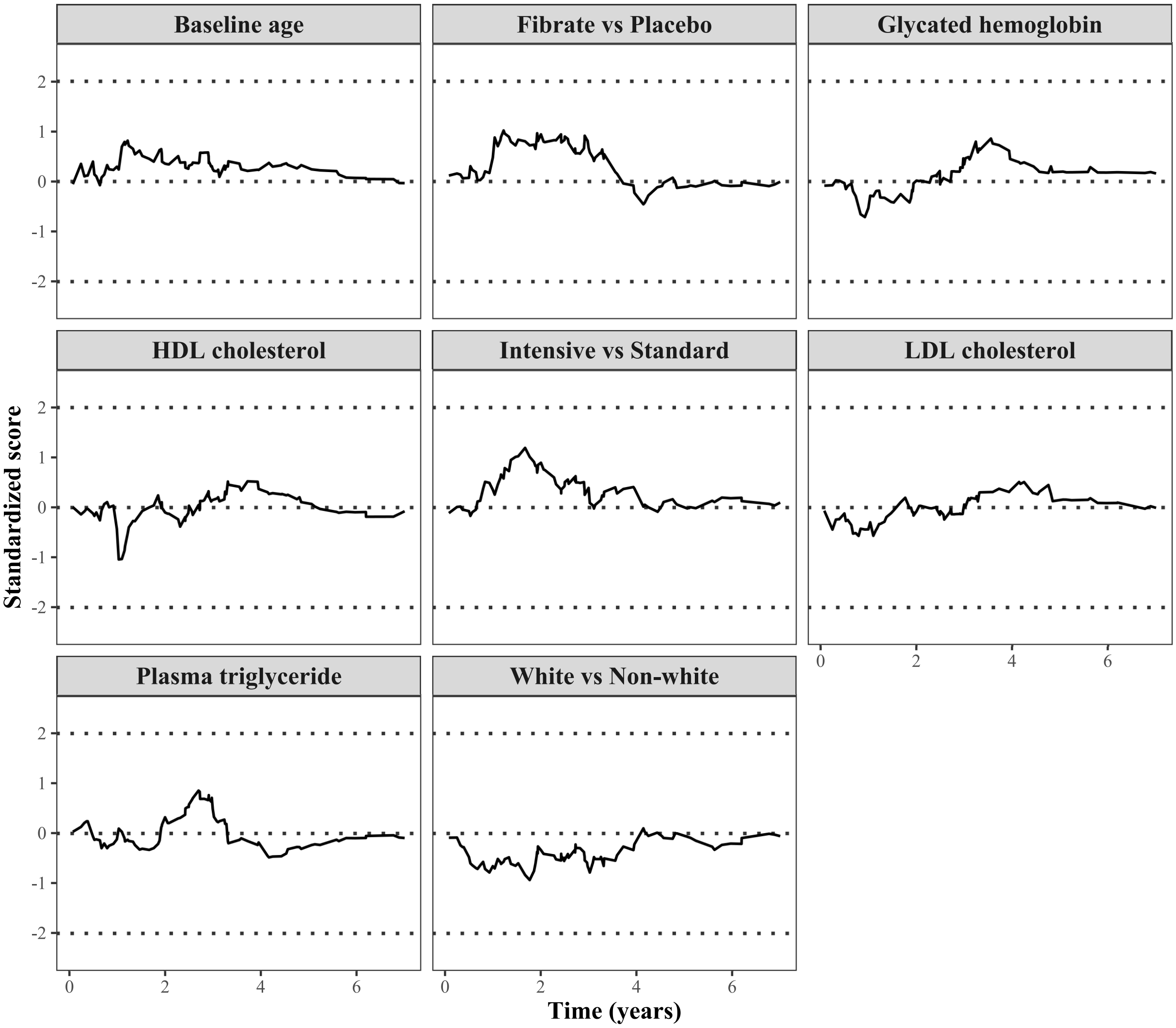

FIGURE S1 Standardized score processes for sex-stratified PW model in Section 5.1.

(P-value 0.277), with delayed all-cause death and reduced number of hospitalizations, compared to those receiving usual care alone.

References

1. Mao L, Kim K, Li Y. On recurrent-event win ratio. *Statistical Methods in Medical Research* 2022: 09622802221084134.

2. Mao L, Wang T. A class of proportional win-fractions regression models for composite outcomes. *Biometrics* 2021; 77(4): 1265–1275.

3. van der Vaart A. *Asymptotic Statistics*. Cambridge: Cambridge University Press . 1998.

4. Hoeffding W. A Class of Statistics With Asymptotically Normal Distributions. *Annals of Mathematical Statistics* 1948; 19: 293–325.

5. O'Connor CM, Whellan DJ, Lee KL, et al. Efficacy and safety of exercise training in patients with chronic heart failure: HF-ACTION randomized controlled trial. *Jama* 2009; 301(14): 1439–1450.

TABLE S1 Baseline features of the ACCORD Lipid study subset in Section 5.2.

Variable	fenofibrate (N=483)	Placebo (N=455)	Overall (N=938)
Age — year	60.3 (57.0 - 65.2)	61.5 (57.6 - 65.9)	60.8 (57.3 - 65.6)
Female — no. (%)	95 (19.7%)	92 (20.2%)	187 (19.9%)
Intensive therapy — no. (%)	250 (51.8%)	221 (48.6%)	471 (50.2%)
Previous cardiovascular history — no. (%)	195 (40.4%)	186 (40.9%)	471 (40.6%)
Race — no. (%)			
White	364 (75.4%)	362 (79.6%)	736 (77.4%)
Black	35 (7.2%)	21 (4.6%)	56 (6.0%)
Hispanic	24 (5.0%)	26 (5.7%)	50 (5.3%)
Other	60 (12.4%)	46 (10.1%)	106 (11.3%)
Glycated hemoglobin — %	8.2 (7.6 - 9.0)	8.3 (7.7 - 8.9)	8.2 (7.6 - 8.)
Plasma cholesterol — mg/dl			
Total	182 (159 - 208)	183 (159 - 212)	182 (159 - 210)
Low-density lipoprotein	93 (74 - 116)	95 (76 - 119)	93 (74.3 - 117)
High-density lipoprotein	30 (27-33)	30 (27-33)	30 (27-33)
Plasma triglyceride —– mg/dl	291 (238 - 376)	276 (232 - 357)	285 (236 - 364.8)
First-event rate — year^{-1}	0.026	0.039	0.032
CV death rate — year^{-1}	0.007	0.014	0.010

Note: Quantitative variables are summarized by median (inter-quartile range) and categorical variables by N(%).

TABLE S2 Etiology-stratified PW regression analysis of the HF-ACTION study.

	Etiology-stratified PW		
Variable	Win ratio	95% confidence interval	P-value
Training vs Usual	1.06	(0.95, 1.19)	0.277
Female vs Male	1.20	(1.05, 1.36)	0.006
Age	0.99	(0.98, 0.99)	<0.001
BMI	0.99	(0.98, 1.00)	0.074

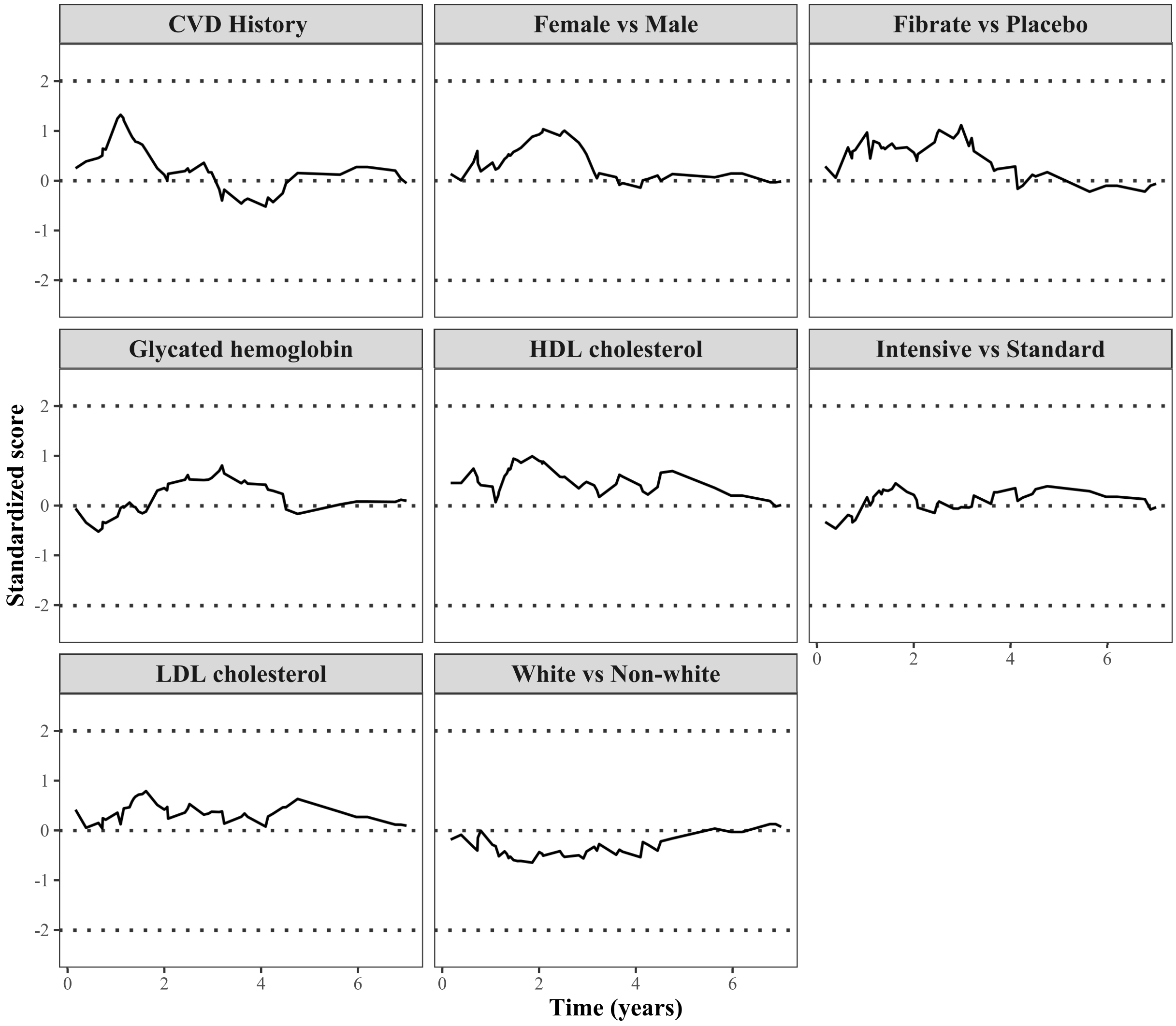

FIGURE S2 Standardized score processes for age-stratified PW model in Section 5.2.

□
